# Thermo-hydro-mechanical response of energy-piled walls under varying wall configurations, pipe layouts, and seepage conditions

**DOI:** 10.1038/s41598-026-42923-z

**Published:** 2026-03-16

**Authors:** Luis Villegas, Guillermo Narsilio, Raul Fuentes

**Affiliations:** 1 Department of Infrastructure Engineering, The University of Melbourne, Melbourne, 3010 Australia; 2 Institute of Geomechanics and Underground Technology, RWTH Aachen University, Aachen, 52074 Germany

**Keywords:** Energy science and technology, Engineering

## Abstract

Energy-piled walls combine earth-retaining and thermal energy-harvesting functions; however, their thermo-mechanical behaviour remains less understood than that of energy foundation piles. This study investigates the coupled thermo-hydro-mechanical response of energy-piled walls using 3D, time-dependent, coupled finite-element models over a six-month heating period, focusing on the effects of wall type, pipe layout, seepage, and thermo-induced pore water pressure. Key findings indicate that: (i) wall type and thermal boundaries influence lateral displacements, with magnitudes remaining below ±2mm under all conditions examined; (ii) the 4U-shaped pipe layout may be preferred over the spiral layout due to generally lower thermo-induced stresses; (iii) seepage enhances heat exchange capacity but introduces wall-slab-seepage interactions that invert bending moment distributions at excavation depth, with behaviour controlled by permeability thresholds ($k \ge {9.09}\times {10}^{-13}{\textrm{m}}^{2}$ for seepage-dominated; $k < {9.09}\times ^{-17}{\textrm{m}}^{2}$ for pore-pressure-dominated behaviour); (iv) peak tensile stresses can exceed concrete capacity, particularly at slab-pile connections, indicating potential localised cracking. The findings are synthesised into a conceptual framework that accounts for these coupled interactions and provides quantitative design guidance across diverse wall configurations, pipe layouts and ground conditions.

## Introduction

Shallow geothermal energy is a renewable energy source proven to be a cost-efficient solution for heating and cooling applications. It levers on the relatively stable temperature of the ground at shallow depths, using it as both a heat sink and source, with heat extraction enabled through a Ground Source Heat Pump (GSHP) and Ground Heat Exchangers (GHE). This technology offers higher efficiency than conventional electrical heating and cooling systems, operating at coefficients of performance around 4–meaning it provides 4kW of thermal energy for every 1kW of electricity consumed– reducing energy consumption by up to 75%^1^. Traditionally, boreholes have been used as GHEs; however, the high initial capital investment required for drilling has driven interest in alternative solutions such as energy piles^2^. Energy piles integrate heat absorber pipes within conventional deep foundation piles, simultaneously transferring structural loads to the ground and functioning as GHEs^1^. Their early use was reported in Austria in the 1980s^1^; however, it was not until the 21 st century that they began to be extensively investigated under various conditions (e.g^3–7^.,). These investigations have improved the understanding of their thermal and thermo-mechanical performance^8^ and underscored their need to be integrated towards sustainable operation of district heating networks^9^, with applications extending to other geotechnical structures such as pavements, tunnels and retaining walls.

The use of energy piles within retaining walls has gained increasing attention as studies have demonstrated their potential to contribute to thermal energy demands^10–12^, while analytical solutions are being developed^13^. Field studies investigating the thermomechanical response of single^1,14–18^ and multiple^19^ energy piles have highlighted the importance of thermo-induced stress along the pile section. Moreover, as the number of closely spaced thermo-active piles increases, their response becomes analogous to a continuous energy (diaphragm) wall^19,20^. In these walls, thermal activation induces larger lateral displacements, resulting in thermo-induced bending moments and shear forces that are worth considering in their engineering design. The magnitude of these effects is highly dependent on thermal boundary conditions, as demonstrated by numerical assessments^21,22^. However, existing field studies on energy-piled walls have been conducted over limited periods, capturing only temporary conditions that may not entirely reflect long-term performance. To address this, numerical studies have been widely employed, particularly for energy diaphragm walls, providing critical insights into their thermal and structural behaviour^21–26^. Nonetheless, key engineering aspects determined during early design stages–such as wall type and pipe layouts–remain underexplored for energy-piled walls. In particular, seepage effects, which have been shown to enhance thermal performance^12^, along with thermo-induced pore water pressures, have been either overlooked or not systematically assessed in numerical studies. These factors are fundamental to developing a comprehensive conceptual framework for the response of energy-piled walls, necessitating further research.

This study aims to deepen the understanding of the thermo-mechanical behaviour of energy-piled walls through a comprehensive parametric investigation that extends beyond existing field and numerical studies. While recent studies, including the authors’ previous work^19,20^, have validated thermal and thermo-mechanical coupling in energy-piled walls, they remain limited to single configurations without hydraulic coupling. Thus, this study extends that validated numerical model to systematically investigate multiple wall types (cantilever, double-propped, and multi-propped), comparative pipe layout effects (4U-shaped versus spiral), and coupled thermo-hydro-mechanical behaviour across six orders of magnitude in permeability. To achieve this, a series of coupled numerical models, integrating fundamental physical processes, are employed to better comprehend the response of this energy geostructure under varying ground site conditions, wall configurations, pipe layouts, and thermal and hydraulic boundary conditions. The findings identify critical permeability thresholds and wall-slab-seepage interactions characteristic of energy-piled walls, providing new insights into the relationship between thermal loads, structural demands, and hydrogeological conditions through a quantitative assessment and a comprehensive conceptual framework for energy-piled walls.

## Methods

### Numerical modelling

#### Overview

The governing equations describing each physical phenomenon are implemented in the finite element software COMSOL Multiphysics V.6.2. The equations are coupled and used under the following assumptions:

- Displacement, velocity, and temperature fields are continuous across all components (i.e., ground, wall, slabs, and connections).
- All materials are homogeneous and isotropic, and their response is elastic under mechanical loading and temperature changes.
- The ground is a continuum, and its phase components are represented by effective (equivalent) properties.
- Transient inertial effects are neglected, and the displacement field of the solids is quasistatic.
- The wall is wished-in-place, and construction effects are neglected.
- Mechanical sign conventions are used, with positive tensile strain and stress.
- Single-phase fluid flow occurs within the ground.
- Unsaturated conditions are not accounted for, with the ground being either fully saturated (voids filled with water) or dry (voids filled with air).
- Heat exchangers are represented by 1D elements with cross-section averaged quantities (velocity, pressure, and temperature fields).
- The fluid within the pipes is incompressible and flows under a turbulent regime.

### Governing equations

#### Heat exchangers, pipe mass flow and heat transfer

The heat exchangers are represented as 1D elements governed by the continuity, momentum and energy conservation principles. The fluid mass flow component involving the mean fluid velocity $\bar{v}_{f, i}$ and pressure $p_f$ fields within the pipes is computed by solving the continuity (Eq. 1) and momentum (Eq. 2) equations.

1 $$\begin{aligned} & \nabla \cdot ( A_p \rho _f \bar{v}_{f, i}) = 0 \end{aligned}$$

2 $$\begin{aligned} & \rho _f \frac{\partial \bar{v}_{f, i}}{\partial t} = -\nabla p_f - f_D \frac{\rho _f}{2 d_h} | \bar{v}_{f, i} | \bar{v}_{f, i} \end{aligned}$$

where $A_p$ is the pipe cross-sectional area, $\rho _f$ is the fluid density, $f_D$ is the Darcy friction factor (computed using Churchill’s^27^ friction model), and $d_h$ is the pipe hydraulic diameter.

The heat exchange between the carrier fluid and its surrounding medium involves solving for the carrier fluid temperature within the pipe $\bar{T}_f$. This uses Eqs. 3 and 4, representing the energy balance accounting for convection-conduction heat transfer within the pipes. The coupling between the pipe fluid and the surrounding medium conductive temperature field (i.e., concrete pile) derives from the energy balance between the heat exchanged through the pipe wall $\dot{q}_p$ and the medium, as described by Eq. 5.

3 $$\begin{aligned} & \rho _f C_{p, f} A_p \frac{\partial \bar{T}_f}{\partial t} + \rho _f C_{p, f} A_p \bar{v}_{f, i} \cdot \nabla \bar{T}_f = \nabla ( A_p \lambda _f \nabla \bar{T}_f) + f_D \frac{\rho _f A_p}{2 d_h} | \bar{v}_{f, i} | \bar{v}_{f, i}^2 + \dot{q}_p \end{aligned}$$

4 $$\begin{aligned} & \dot{q}_p = f({T}_{\text {pipe wall}}, \bar{T}_f) \end{aligned}$$

5 $$\begin{aligned} & \rho _m C_{p, m} \frac{\partial {T}_m}{\partial t} = \nabla (\lambda _m \nabla {T}_m) \end{aligned}$$

where $\rho$, $C_p$, and $\lambda$ denote density, heat capacity, and thermal conductivity, with subscripts *f* and *m* referring to the fluid and medium, respectively. ${T}_{\text {pipe wall}}$ represents the external pipe wall temperature.

#### Ground, fluid mass flow and heat transfer

Darcy’s law describes single-phase fluid flow in a porous medium (groundwater flow), computing the fluid velocity ${v}_{m, f}$ and pressure $p_{m_f}$ fields using continuity (Eq. 6) and momentum (Eq. 8) equations:

6 $$\begin{aligned} {v}_{m, f} = -\frac{k}{\mu _{m_f}} (\nabla p_{m_f} - \rho _{m_f} g z), \end{aligned}$$

where,

7 $$\begin{aligned} & k = k_h \frac{\mu _{m_f}}{\rho _{m_f} g}, \end{aligned}$$

8 $$\begin{aligned} & \nabla \cdot (\rho _{m_f} {v}_{m, f}) = - \frac{\partial }{\partial t} (\rho _{m_f} n). \end{aligned}$$

where $k$ is the porous medium (e.g., ground) intrinsic permeability, $k_h$ is the hydraulic conductivity, $\mu _{m_f}$ is the fluid dynamic viscosity, $\rho _{m_f}$ is the fluid density, $g$ is the gravitational acceleration, and *n* is the porosity of the medium.

The porous nature of the ground affects heat transfer characteristics. In addition to its thermal properties, groundwater flow must be considered due to convection effects. In this case, the previously introduced heat transfer equation for conduction (recall Eq. 5) is extended to a more general form in Eq. 9, accounting for the effective ground properties, estimated by volume averaging the ground solid and fluid components, as shown in Eqs. 11 and 12.

9 $$\begin{aligned} (\rho C_p)_{eff} \frac{\partial {T}_m}{\partial t} + (\rho _{m_f} C_{p, m_f} {v}_{m, f}) \cdot \nabla {T}_m + \nabla \cdot q_m = - \frac{\partial }{\partial t} (\rho _{m_f} n), \end{aligned}$$

where,

10 $$\begin{aligned} & q_m = -\lambda _{eff} \nabla {T}_m, \end{aligned}$$

11 $$\begin{aligned} & (\rho C_p)_{eff} = (1-n) \rho _{m_{\text {s}}} C_{p, m_{\text {s}}} + n \rho _{m_f} C_{p, m_f} \end{aligned}$$

12 $$\begin{aligned} & \lambda _{eff} = (1-n) \lambda _{m_{\text {s}}} + n \lambda _{m_f} \end{aligned}$$

where $\rho _{m_{\text {s}}}$ and $C_{p, m_{\text {s}}}$ are the density and specific heat capacity of the solids, $\rho _{m_f}$ and $C_{p, m_f}$ are the density and specific heat capacity of the fluids, and $\lambda _{m_{\text {s}}}$ and $\lambda _{m_f}$ are the thermal conductivities of the solids and fluids, respectively.

#### Ground and wall, momentum and fluid mass conservation

The momentum equilibrium in porous media (ground) is expressed as:

13 $$\begin{aligned} \nabla \cdot \sigma _{ij} - \nabla p_f + \rho g_i = 0, \end{aligned}$$

or equivalently,

14 $$\begin{aligned} \nabla \cdot [C_{ijkl} : \varepsilon _{kl}(u_{s}, T_m)] - \nabla p_f + \rho g_i = 0, \end{aligned}$$

The mass conservation equation of the fluid phase is given by:

15 $$\begin{aligned} \nabla \cdot {v}_{m, s}+ \nabla \cdot {v}_{m, f} + \frac{1}{Q} \frac{\partial p_f}{\partial t} - \beta _{t} \frac{\partial T_m}{\partial t} = 0 \end{aligned}$$

where,

16 $$\begin{aligned} & \frac{1}{Q} = \frac{1 - n}{{K_{m_\text {s}}}} +\frac{n}{K_{m_f}} \end{aligned}$$

17 $$\begin{aligned} & \beta _{t}= (1 - n) \beta _{m_{\text {s}}} + n \beta _{m_f} \end{aligned}$$

where $\sigma _{ij}$ is the stress tensor, $u_{s}$ is the solids displacement field, ${v}_{m, s}$ is the solids velocity, $\beta _{m_{\text {s}}}$ and $\beta _{m_f}$ are the volumetric thermal expansion coefficients of the solid and fluid components, and ${K_{m_\text {s}}}$ and $K_{m_f}$ are the elastic bulk moduli of the solid and fluid components.

The validation of the thermal and thermo-mechanical numerical coupling -comprising pipe mass flow and heat transfer, solid heat transfer and the conservation of mass and momentum in the energy piled walls- is presented in Villegas et al.^19^. This paper introduces an additional component: the thermo-hydro-mechanical response of the porous medium, described by Eqs. 6, 9, and 15. For this component, a comparison between the numerically predicted and analytically computed thermo-induced pore water pressure is provided in Supplementary Material as proposed by Cui^28^.

#### Models description

#### Energy piled walls

**Fig. 1:**
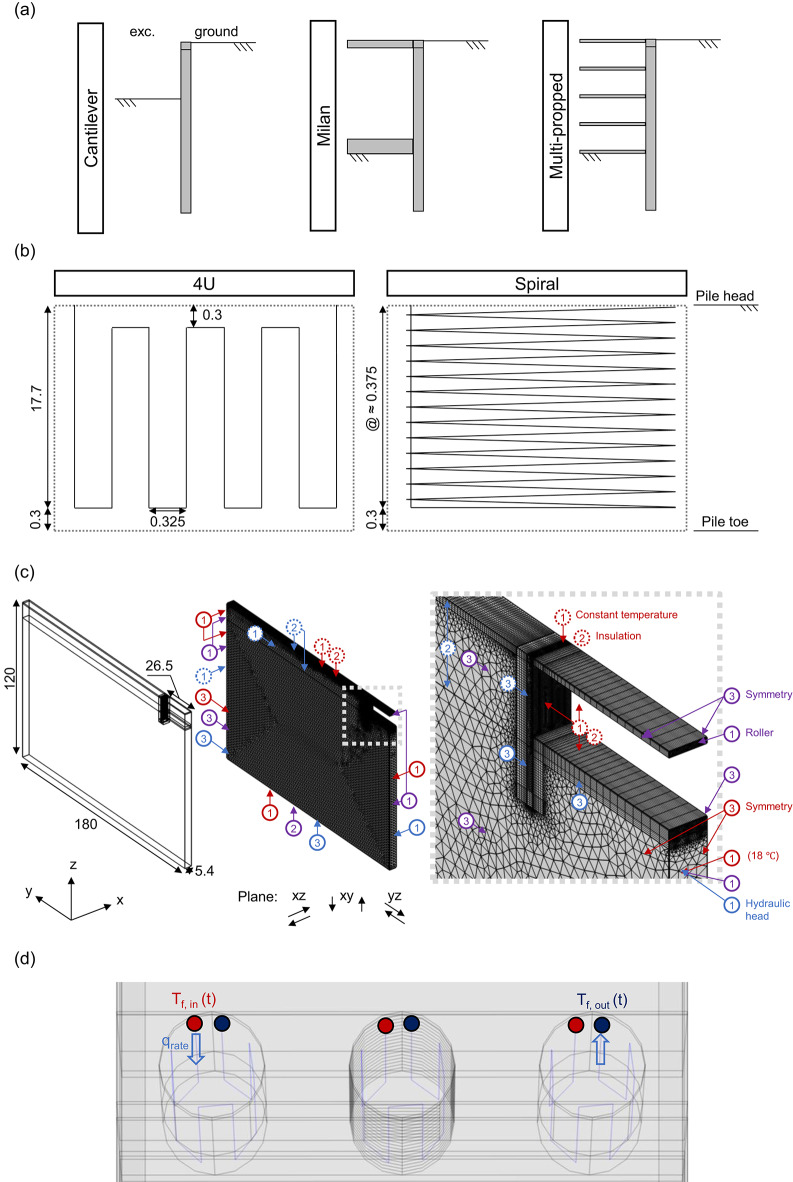
Energy piled-wall models. (**a**) Wall types; (**b**) pipe layouts; (**c**) numerical model and boundary conditions; (**d**) pipes. All units are in meters.

*Piled walls:* this study uses typical piled wall configurations for excavation depths of 6 and 12 m in medium to rigid ground conditions. The piles are assumed to be made of concrete and to have a constant length of 18 m, a diameter of 1.0m, and a centre-to-centre spacing of 1.8m. Three piled wall configurations are considered: a cantilever wall, a double-slab propped wall, and a multi-slab propped wall, as schematically shown in Fig. 1 (a). All piles are connected by a continuous capping beam with a height of 0.8m at the top and a lagging wall of 0.15m thickness along the depth. For the slab-propped walls, the slabs in the double-propped wall (referred to as the Milan wall) have varying thicknesses, with the upper and bottom slabs being 0.8m and 1.5m thick, respectively. In contrast, the multi-propped wall uses slabs of constant thickness, each 0.3m. All slab-propped wall configurations assume rigid connections between the wall and the slabs.

*Pipe layouts:* the absorber High-Density Polyethylene (HDPE) pipes, each with an outer diameter of 25 mm and a thickness of 2.3mm, are arranged around the perimeter of the piles, with a single inlet and outlet at the pile head. The pipes are embedded within the pile and have a 75 mm concrete cover from the pile surface. The layout consists of either 4 U-shaped HDPE pipe loops or a spiral configuration, with the same total length of the pipes across both layouts. The pipes are symmetrically distributed for the 4 U-shaped loops, with a separation-to-shank distance of 1, as recommended for optimal thermal performance in energy piles^29^. The loops are spaced 0.3m from the pile head and toe. For the spiral loops, the pitch between the loops is approximately 0.375m. More details of the pipe layouts are shown in Fig. 1 (b).

*Seepage:* groundwater flow is induced by the hydraulic gradient between the retained and excavated sides, with the intrinsic permeability held constant. No seepage occurs when the water level is the same on both sides of the wall, at the excavation level.

#### Numerical models

*Materials:* the material properties used in the numerical models are summarised in Table 1. These values, which are adopted from studies on energy walls^11,12,21,26^, represent the ground, concrete, HDPE pipes, water, and air. They serve as baseline parameters for the subsequent parametric study detailed in Parametric study Section.

**Table 1:** Material properties.

Material/Property	Ground	Concrete	HDPE (pipe)	Water^*^
$$\rho$$$$[{\textrm{kgm}}^{-3}]$$	2350	2373	-	998.2
*E*$[{\textrm{MPa}}]$	78	${30}\times {10}^{3}$	-	-
$$\nu$$$$[-]$$	0.3	0.2	-	-
*K*$[{\textrm{MPa}}]$	65	${16.6}\times {10}^{3}$	-	${2.2}\times {10}^{3}$
$$\alpha$$$$[\upmu \varepsilon /{\textrm{K}}]$$	20	10	-	69
*k*$[{\textrm{m}}^{2}]$	${9.09}\times {10}^{-12}$	-	-	-
$$\lambda$$$$[{\textrm{Wm}}^{-1}{\textrm{K}}^{-1}]$$	1.70/2.00^**^	1.80	0.40	0.58
$$C_p$$$$[{\textrm{Jkg}}^{-1}{\textrm{K}}^{-1}]$$	780.0	850.0	-	4186.9

*Values with reference to ${20}^{\circ }{\textrm{C}}$.

**with *n* = 0.5, $\lambda _{m_{\text {s}}}$ = 3.42, and either air $\lambda _{m_{\text {a}}}$ = 0.02 or water $\lambda _{m_{\text {w}}}$ = 0.58 in Eq. 12.

*Initial values and boundary conditions:* a steady state analysis is performed to initialise transient studies. The steady-state analysis considers initial displacement and velocity fields for the porous medium equal to zero, while the temperature field is initialised at a constant value of ${18}^{\circ }{\textrm{C}}$, representative of typical undisturbed ground conditions in Melbourne, Australia^30^. The initial equilibrium of the porous medium displacement, fluid velocity, and temperature fields is reached under the boundary conditions shown in Fig. 1 (c).

Fig. 1 (c) illustrates the assigned boundary conditions, categorised as mechanical (purple), thermal (red), and hydraulic (blue). Assigned mechanical boundaries restrict perpendicular displacements ($\textcircled {1}$, roller boundary) on the sides (xz-planes) and in both vertical and lateral displacements ($\textcircled {2}$, pinned boundary) at the model base (xy-plane). Symmetry $\textcircled {3}$ (equivalent to a roller) is imposed on the remaining vertical faces (yz-planes). No surface constraints are applied to the xy-plane or the exposed wall surface, allowing free movement.

Thermal boundaries include constant temperature $\textcircled {1}$ and perfect insulation $\textcircled {2}$ (zero heat flux). A constant temperature equal to the initial ${18}^{\circ }{\textrm{C}}$ is assigned at the bottom (xy-plane), and the vertical side surfaces (xz-planes) represent far-field conditions. Symmetry $\textcircled {3}$ is imposed on the vertical faces (yz-planes). The remaining exposed surfaces, representing the excavation and surroundings, can have a constant temperature or a perfect insulation condition. These idealised conditions bound the expected thermo-mechanical response, consistent with previous numerical studies^21,22^.

Hydraulic boundaries consist of constant hydraulic heads $\textcircled {1}$ assigned to the upstream and downstream xz- and yz-planes. Null pressure $\textcircled {2}$ is applied to the upper xy-plane on the retained side when the hydraulic gradient is at its maximum and to the bottom retained and excavation sides when it is null. No flow $\textcircled {3}$ is allowed (impervious) on the remaining exposed surfaces. In all cases, the downstream hydraulic head remains constant across all analyses, equal to the position head. The upstream head is set equal to the downstream head plus the excavation depth (i.e., 6 m for cantilevers and 12 m for other wall types -further detailed in Parametric study Section).

At initial thermal equilibrium, when the GHE is not operating, the velocity of the circulating heat carrier fluid (i.e., water within the absorber pipes) is set to zero, the pressure at the outlets is atmospheric (101325Pa), and the initial temperature equates the undisturbed ground temperature.

Figure 1 (d) illustrates the 4U shape loops pipe layout and boundary conditions, with all piles operating in parallel. The carrier fluid circulates within the pipes at a constant flow rate ($q_\text {rate}$) of ${5.5}{\textrm{L}}{\textrm{min}}^{-1}$ ($\bar{v}_{f,i}$ = ${0.28}{\textrm{m s}}^{-1}$), ensuring a turbulent regime and efficient heat transfer^1^. As indicated in the figure, a time-dependent temperature function is prescribed at the pile inlet ($\bar{T}_{f,\text {in} (t)}$), as described by Eq. 18.

18 $$\begin{aligned} \bar{T}_{f,\text {in} (t)} = \bar{T}_{f,\text {out} (t)} - \frac{Q_{(t)}}{A_p \bar{v}_{f,i} \rho _f C_{p,f}} \end{aligned}$$

where $\bar{T}_{f,\text {in} (t)}$ and $\bar{T}_{f,\text {out} (t)}$ are the time-dependent average temperatures of the carrier fluid at the pipe inlets and outlets, and $Q_{(t)}$ is the time-dependent thermal load per pile.

*Thermal load:* the thermal load per pile is defined over six months of continuous heat rejection (i.e., cooling the building), with all piles activated and in optimal operational conditions. Optimal thermal performance is achieved when the average carrier fluid temperature ($T_\text {ave}$) within the pipes is approximately ${40}^{\circ }{\textrm{C}}$^12,31^. The six-month duration reflects a seasonally balanced operational regime, assuming six months of heating and six months of cooling.

Under these conditions, the thermal load is examined through a representative section of the wall, as described in Supplementary Material, and across all conditions outlined in Parametric study Section. Throughout the analysis, the average fluid temperature of ${40 \pm 0.1}^{\circ }{\textrm{C}}$ is maintained.

*Model dimensions and mesh discretisation:* model dimensions and mesh discretisation were defined to avoid boundary effects and ensure numerical accuracy. The absorber pipes are discretised using linear elements, while the surrounding domains use a graded mesh. Finer tetrahedral elements are applied to the piled wall and immediate surrounding ground, and finer cubic elements are used for the lateral supports and the ground near the wall and surface; coarser tetrahedral elements are assigned to the remaining domains to optimise computational efficiency.

A mesh sensitivity analysis with progressively refined meshes showed negligible changes in the solved fields (temperature and displacement) between the finest mesh and the adopted mesh. The selected maximum element sizes were 0.6m for the 1D pipe elements and 0.25m for the wall and refined near-field domains.

The final model dimensions and discretisation scheme are illustrated in Fig. 1 (c) for the Milan wall, with consistent dimensions across all models. Additional details are provided in the Supplementary Material.

#### Parametric study

The parametric study examines key variables related to ground properties and construction configurations, as summarised in Table 2. Ground properties (mechanical, hydraulic, and thermal) are selected to represent a broad spectrum of ground conditions, ranging from soft to rigid materials. To systematically assess the sensitivity of energy-piled wall response to individual parameters, both characteristic values and limiting cases spanning several orders of magnitude are included. Construction factors, including wall type and pipe layout, are analysed under realistic configurations, while water level position and thermal boundary conditions are varied over broader ranges representing extreme scenarios. Results are compared against a baseline case representing a medium-to-rigid ground condition (as introduced in the Models description Section). Characteristic values of the baseline case are highlighted in bold in Table 2.

**Table 2:** Parametric study.

Variable	Property	Value
Ground	*E*, [MPa]	7.8, **78.0**, 780.0
	$\alpha$,$[\upmu \varepsilon /{{K}}]$	0, **20**,40
	*k*, [${\textrm{m}}^{2}$]	(${\textbf {9.09}}\times {10}^{-13}, {9.09}\times {10}^{-19}$)
	$\lambda _{m_{\text {s}}}$*, [${\textrm{Wm}}^{-1}{\textrm{K}}^{-1}$]	1.42, **3.42**, 5.42
Construction	Wall type	cantilever, Milan, multi-propped slabs wall
	Pipe layout	**4U-shape**, spiral
	Water level	top, **bottom**
	Thermal boundary	constant temperature, insulation

Parameters such as density, Poisson’s ratio, porosity, and heat capacity are kept constant, and construction effects are not considered. Although some parameters in Table 2 are interrelated, independent variation of each parameter -while fixing all others at reference values- enables isolation of key mechanisms and assessment of individual impacts on structural demands and serviceability.

### Engineering analysis

The geothermal potential and engineering implications are analysed at the end of six months of heating (cooling the building). The geothermal potential is evaluated by computing the thermal load per pile, *Q*, through Eq. 18, in the form:

19 $$\begin{aligned} Q_{(t)} = \left( \bar{T}_{f,\text {out} (t)} - \bar{T}_{f,\text {in} (t)} \right) A_p \bar{v}_{f,i} \rho _f C_{p,f} \end{aligned}$$

Thermo-induced shear forces and lateral displacements are evaluated at the pile centre using the stress on the xy plane and the displacement component in the y-direction. Bending moments are computed from the axial strain in the z-direction on two opposite sides of the pile (i.e., ground and excavation), measured at a distance of 75 mm (pipe cover) from the pile surface. These are determined using Eq. 20, a method previously applied in experimental and numerical studies^15,32,33^.

20 $$\begin{aligned} M = \frac{E I (\varepsilon _{\text {ground}} - \varepsilon _{\text {excavation}})}{h} \end{aligned}$$

where *M* is the bending moment, *E* the concrete elasticity modulus, *I* the second moment of inertia, *h* the distance between evaluation points (i.e., pile diameter minus two times pipe cover), $\varepsilon _{\text {ground}}$ the vertical component of the thermo-induced strain at the ground side, and $\varepsilon _{\text {excavation}}$ the vertical component of the thermo-induced strain at the excavation side.

## Results and discussion

### Geothermal performance

The effect of loop layout on thermal performance is omitted, as the total pipe length remains the same for both 4U-shaped loops and spiral configurations (see discussions by Makasis et al.^10,34^). The thermal load per pile, *Q*, is computed using Eq. 19. Table 3 summarises the thermal loads per pile (additional details are provided in Supplementary Material).

**Table 3:** Thermal loads. (CTemp): Constant temperature, (Insu): Insulation.

Wall type	Thermal boundary	Q, [W]				Water level
		$\lambda _{m_{\text {s}}},$	1.42	3.42	5.42	top/bottom
Cantilever	(CTemp) (Insu)		1555	1890	2135	bottom
			470	645	780	
			-	2910	-	top
			-	1405	-	
Milan	(CTemp) (Insu)		2210	2560	2800	bottom
			372	505	605	
			-	3120	-	top
			-	920	-	
Multi-propped	(CTemp) (Insu)		2400	2770	3045	bottom
			360	495	595	
			-	3375	-	top
			-	910	-	

As shown in Table 3, cantilever walls exhibit the highest thermal performance under perfect insulation, but the lowest under constant temperature conditions. In the absence of groundwater flow, differences between wall configurations arise in the conduction term described by the thermal properties and boundary conditions. Under constant temperature (${18}^{\circ }{\textrm{C}}$), slabs and exposed wall surfaces serve as more effective heat sinks than the ground, particularly for thinner slabs, as in multi-propped walls. Under perfect insulation, the cantilever wall outperforms others due to its longer embedded pile section, reduced exposed area, and larger ground volume for heat exchanging.

In the presence of groundwater flow, the thermal load per pile increases, driven by convection. Average seepage velocities of 0.16, and ${0.27}{\textrm{m d}}^{-1}$ around the piles result in thermal load increments of approximately 54, and 22% under constant temperature, and 118, 82, and 84% under perfect insulation for the cantilever, Milan, and multi-propped walls, respectively. With the thermal loads established for all wall configurations and ground conditions (Tables 3 and 4), the following sections examine how these thermal conditions influence the thermo-mechanical and thermo-hydro-mechanical response, with particular emphasis on structural demands and design implications.

### Thermo-mechanical engineering performance

#### Thermo-mechanical response

*Wall type*

The influence of wall type is analysed in two stages: thermo-induced lateral displacements under varying ground conditions (i.e., solids thermal conductivity, linear coefficient of thermal expansion, and elasticity modulus - values in Table 2), along with thermal boundaries and matching thermal loads - values in Table 3; followed by thermo induced structural demands (i.e., shear forces and bending moments) under the most critical ground conditions.

**Fig. 2:**
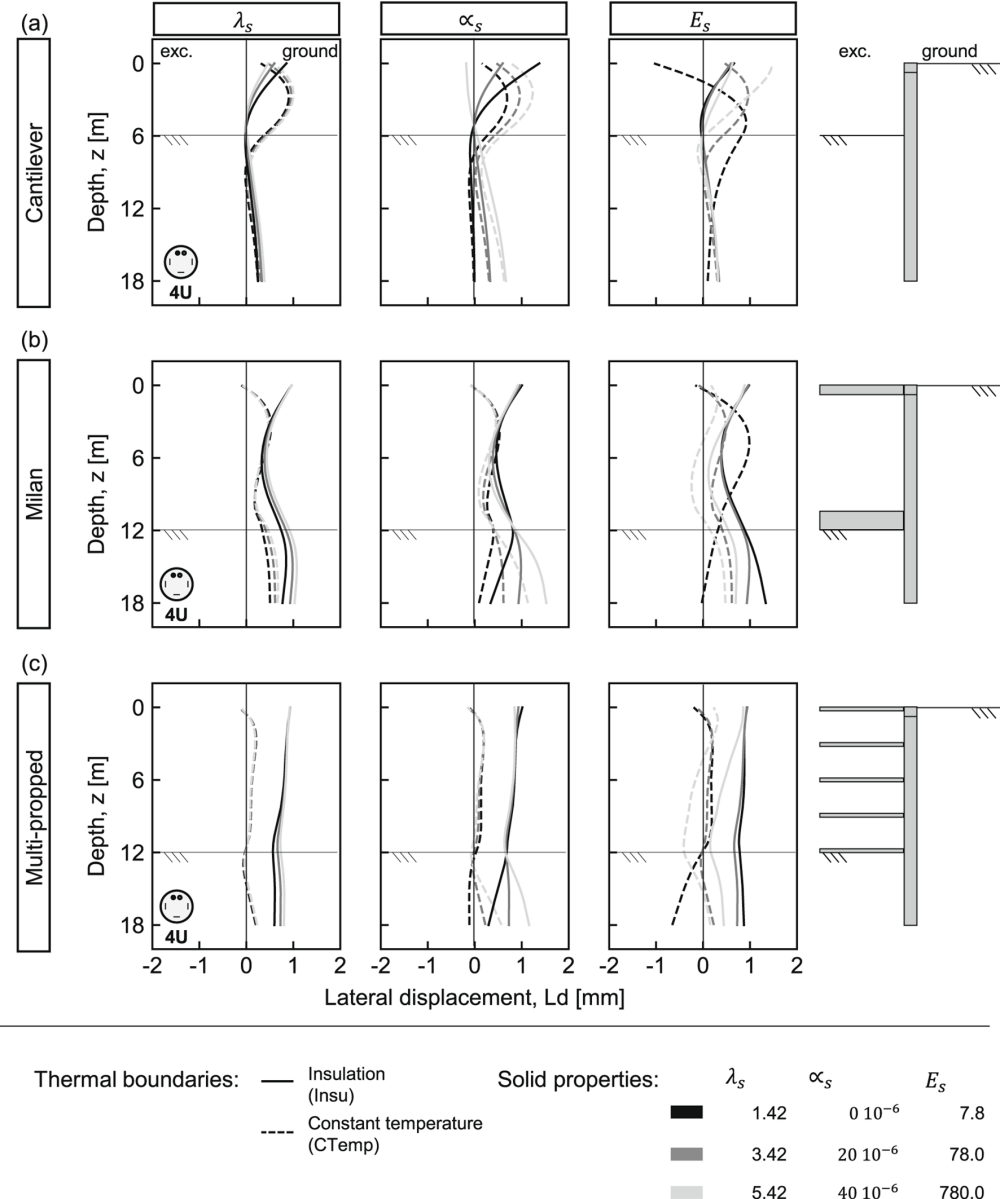
Thermo-induced lateral displacements ($L_d$) in different pile walls under variable ground and thermal boundary conditions. (**a**) Cantilever, (**b**) Milan, and (**c**) multi-propped walls. Continuous and dashed lines represent insulation (Insu) and constant temperature (CTemp), respectively. Line shading indicates parameter magnitude: darkest = lowest values; lightest = highest values.

Figure 2 shows thermo-induced lateral displacements ($L_d$) after six months of continuous heating. In each case, one ground parameter is varied while the others are fixed at their reference (bold) values. Dashed and continuous lines represent constant temperature and perfect insulation, respectively. Dark lines correspond to the lowest parameter (low conductivity/expansion/stiffness), and light grey lines represent the highest values.

The results indicate that the deformation is primarily influenced by thermal boundaries^21,22^, wall type, and ground properties. Under perfect insulation, pile-slab interaction governs displacement as heated slabs push piles toward the ground, causing the embedded pile section to pivot around the bottom slab. Under constant temperature, an opposite curvature develops with lower displacement magnitudes for propped walls, compared to the cantilever. Notably, variations in the elastic modulus result in the largest dispersion of lateral displacements, highlighting it as the most influential ground property, a trend also reported in multi-propped energy diaphragm walls^21^. In all cases, lateral displacements remain below ±2mm, overall suggesting minimal impact on typical engineering applications.

**Fig. 3:**
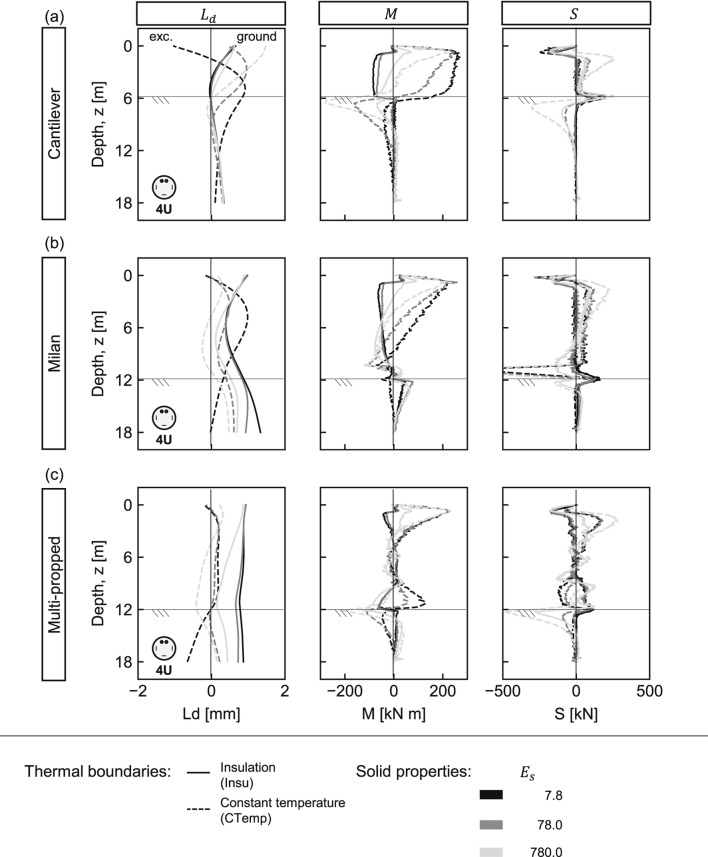
Thermo-induced lateral displacements ($L_d$), bending moments (*M*), and shear forces (*S*) in different pile walls under variable ground stiffness and thermal boundary conditions. (**a**) Cantilever, (**b**) Milan, and (**c**) multi-propped walls. Continuous and dashed lines represent insulation (Insu) and constant temperature (CTemp), respectively. Line shading indicates ground stiffness: darkest = softest; lightest = stiffest.

Figure 3 illustrates induced bending moments (*M*) and shear forces (*S*) under varying ground elastic modulus. Larger structural demands generally develop under constant temperature^21,22^, in stiffer ground, and particularly located near the pile head and at excavation depth. Pile head bending moments are comparable across configurations, with notable transitions from positive to negative moments at the excavation level under constant temperature, and from negative to positive under insulation, for the Milan and multi-propped walls. Shear forces are most pronounced in the Milan walls at pile-bottom slab connections due to high slab stiffness. Maximum values should be carefully assessed, particularly near the pile head, at the excavation depth, and at lateral supports connection. A general maximum-value approach is advisable in structural design; for instance, the results suggest that an additional bending moment of approximately 200KN m per pile should be accounted for along the entire pile length.

*Pipe configuration*

The assessment of pipe configuration effects builds on the findings from the Wall type Section. Accordingly, only variations in ground elasticity modulus are considered, while the spiral pipe configuration is adopted.

**Fig. 4:**
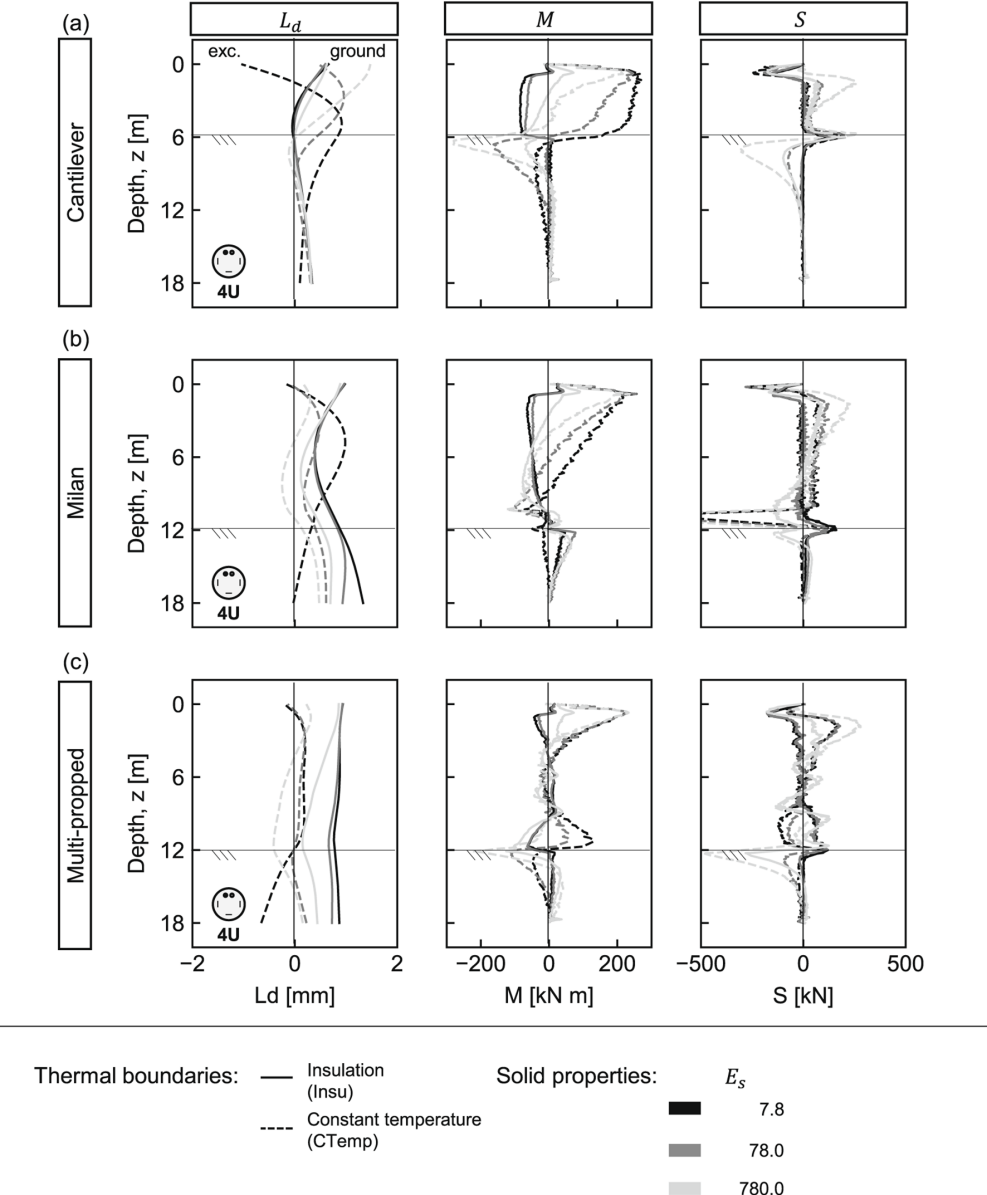
Spiral pipe layout: thermo-induced lateral displacements ($L_d$), bending moments (*M*), and shear forces (*S*) in different pile walls under variable ground stiffness and thermal boundary conditions. (**a**) Cantilever, (**b**) Milan, and (**c**) multi-propped walls. Continuous and dashed lines represent insulation (Insu) and constant temperature (CTemp), respectively. Line shading indicates ground stiffness: darkest = softest; lightest = stiffest.

Figure 4 presents the thermo-mechanical response. In general, minimal differences are observed compared to the 4U-shaped loops (Fig. 3). An oscillatory pattern in bending moment profiles arises from strain measurement locations coinciding with pipe positions. Overall, the most noticeable effects appear in the bending moments, particularly under perfect insulation conditions, where the spiral layout produces slightly lower mean values than the 4U-shaped loops but without significance. In all cases, the largest demands result from the constant temperature conditions. Therefore, it is worth further examination to better understand the influence of pipe layout on the thermo-mechanical response.

**Fig. 5:**
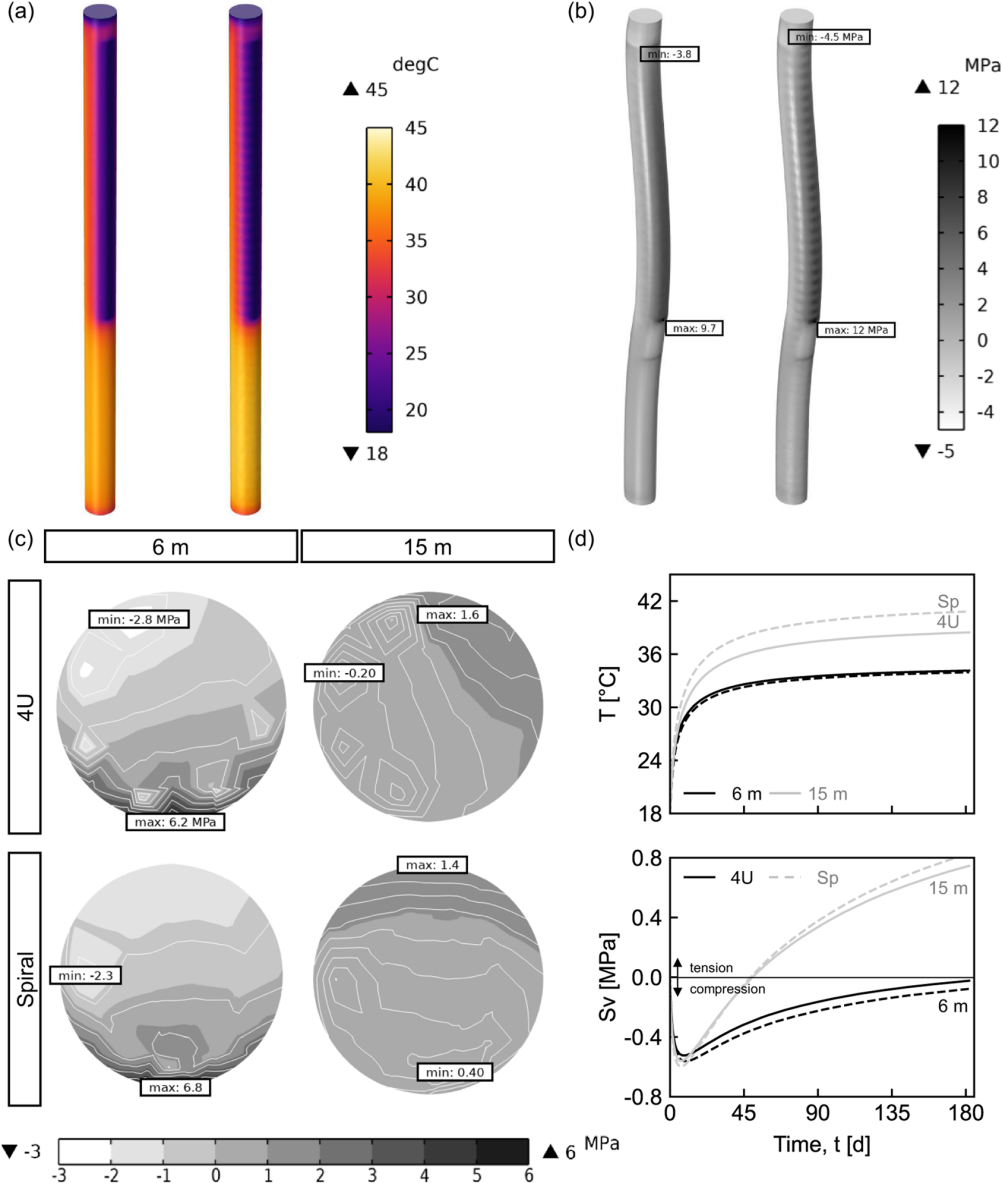
Temperature and vertical thermo-induced stress fields for different pipe layouts in Milan walls under constant temperature boundary conditions in the stiffest ground (*E* = 780 MPa). (**a**) Temperature distribution; (**b**) vertical thermo-induced stress ($S_v$) field; (**c**) vertical thermo-induced stress profiles at 6 m and 15 m depth (horizontal axis scaled by 1000); (**d**) volume-averaged temperature and vertical thermo-induced stress over time at 6 m and 15 m depth. Pipe layouts: 4U (4U-shaped loops), Sp (spiral). Results for other ground conditions and all wall types are provided in Supplementary Material.

Figure 5 compares pipe layouts in the Milan wall under the stiffest (*E* = 780MPa) ground conditions after six months of heating. The temperature distribution in Fig. 5 (a) shows minimal differences in the exposed sections of both configurations, but the spiral configuration exhibits higher temperatures in the embedded section due to longer return paths (i.e., one-third of the total pipe length vs. one-third with two legs). The vertical thermo-induced stress field is shown in Fig. 5 (b). In this case, variations are larger in the spiral layout (16.5MPa range) compared to the 4U-shaped loops (13.5MPa range), with extreme values at slab connections (coinciding with the maximum structural demands, recall figures 3 and 4). At these locations, peak compressive stresses (4.5MPa) remain well below concrete capacity(21MPa to 40MPa). However, peak tensile stresses (12MPa) exceed concrete tensile strength (2MPa to 4MPa), indicating potential cracking at slab–pile connections. While tensile forces are transferred to the reinforcement post-cracking, additional reinforcement or crack-control measures should be considered at these locations.

Figure 5 (c) further examines vertical stresses at 6 and 15 m depth (from the pile head to toe), representing the exposed and embedded sections, respectively. The results indicate that the minimum (negative values -compressive) stress values coincide with the heat injection pipe (inlet), consistent with findings in energy piles^35^ and walls^20,24^. Conversely, the maximum (positive values -tensile) stresses shift toward the excavation side in the upper section and toward the ground in the embedded part. This behaviour is driven by thermal gradients^36^ within the pile. As previously noted, these localised tensile stresses may exceed concrete capacity, particularly near the reinforcement cover at the excavation side, reinforcing the need for additional reinforcement or crack-control provisions, beyond the connections to the exposed pile surface.

Figure 5 (d) presents the transient changes of the volume-averaged temperature and vertical stress within a 2 m segment (1m above and below the representative depth) for both pipe layouts. Maximum compressive stresses occur around 7 and 17 days at 15 and 6 m depth, respectively, gradually becoming more tensile as the ground thermally engages. This behaviour arises from the ground stiffness and the high thermal expansion coefficient (higher than that of concrete) considered in this study, representing an extreme scenario (further details in Supplementary Material). Beyond localised stress points around pipes, no significant differences exist between layouts. For the conditions analysed, with the inlet positioned toward the ground and pipe loops connected in series, reduced stress variations are noted in the 4U-shaped loops, suggesting this configuration may be preferred for reducing thermo-induced effects.

#### Thermo-hydro-mechanical response

The preceding discussions establish the thermo-mechanical response baseline, distinguishing between wall type, ground condition, and pipe configuration effects. This section investigates how seepage and thermo-induced pore pressure modulate structural demands across wall configurations.

*Seepage and pore pressure effects*

Groundwater flow enhances thermal performance, with thermal power, in some cases, doubling (recall the Geothermal performance Section -Table 3), providing benefits for managing unbalanced thermal loads over the long term^12^. Given these advantages and the necessity of accounting for thermo-induced pore water pressures^26^, this study evaluates the response across intrinsic permeabilities representing materials from sands to rocks^37–40^, detailed in Table 2, and with the water level at the retained side surface.

**Fig. 6:**
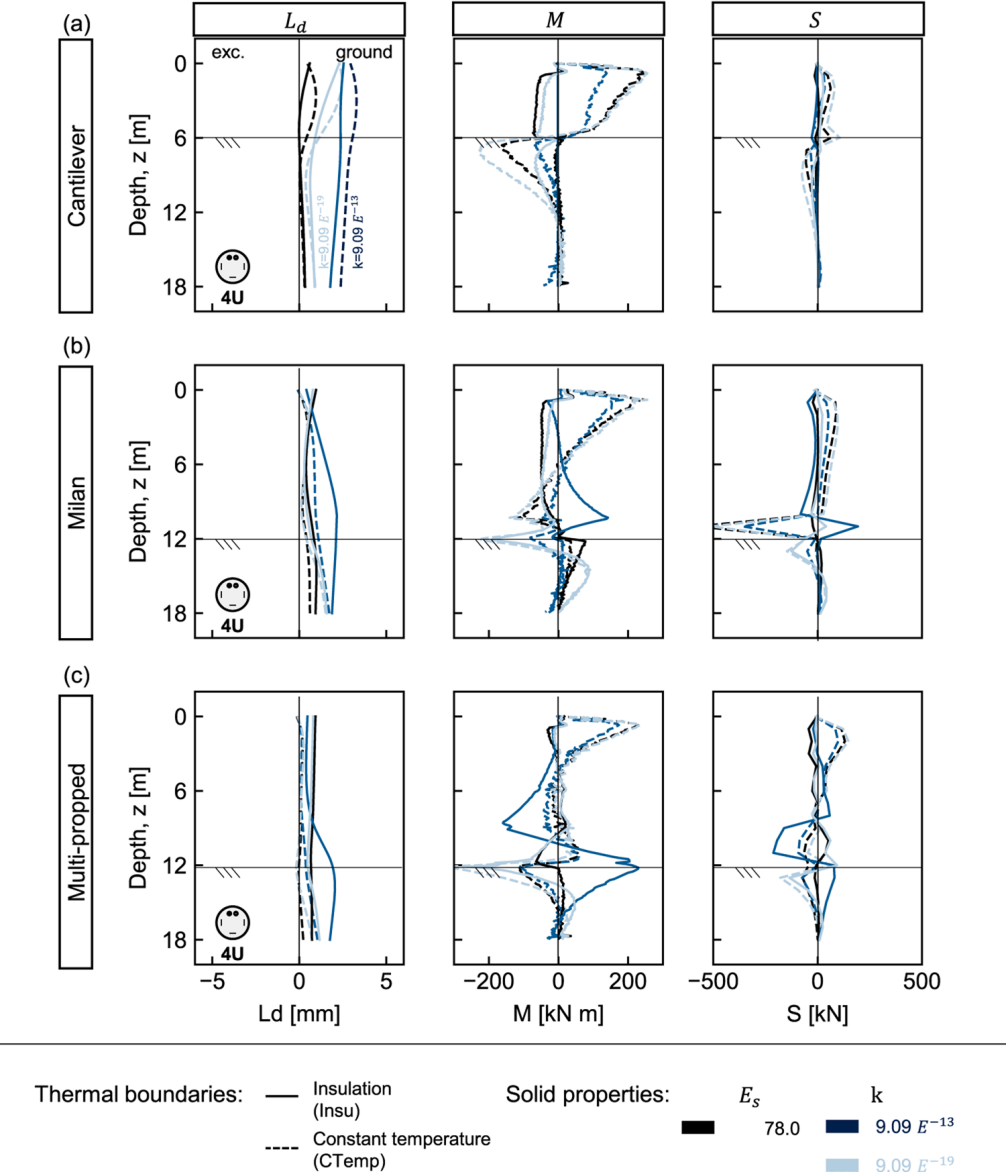
Thermo-hydro-induced lateral displacements ($L_d$), bending moments (*M*), and shear forces (*S*) in different pile walls under varying seepage, intrinsic permeability, and thermal boundary conditions. (**a**) Cantilever, (**b**) Milan, and (**c**) multi-propped walls. Continuous and dashed lines represent insulation (Insu) and constant temperature (CTemp) boundaries, respectively. Line colours: black = baseline case (no hydraulic gradient); dark blue = high permeability ($k = {9.09}\times {\textrm{E}}^{-13}{\textrm{m}}^{2}$); light blue = low permeability ($k = {9.09}\times {\textrm{E}}^{-19}{\textrm{m}}^{2}$).

Figure 6 illustrates seepage and pore water pressure impacts compared against the baseline (black lines–no hydraulic head) and subject to the same thermal load. Under high permeability (dark blue lines), hydrostatic pressure and groundwater flow increase lateral displacements and structural demands. For the Milan and multi-propped walls, the hydraulic pressure and seepage affect the deformed shape through wall-slab interaction. As groundwater exchanges heat with the ground and slabs, thermal expansion induces large shear forces and bending moments at the bottom slab connection, increasing lateral displacements toward the ground. In the cantilever walls, a larger ground volume in front of the embedded pile section is thermally engaged, thereby increasing the magnitudes of displacement.

Under low permeability (light blue lines), lateral displacements decrease relative to high-permeability cases, closely resembling those without groundwater flow. However, the embedded pile section tends to pivot around the bottom slab, leading to higher bending moments and shear forces than the baseline, suggesting that the pore water pressure development increases structural demands.

Extended analysis identifies permeability thresholds distinguishing seepage from pore pressure dominance. In this assessment, the piled wall is thermo-activated at its optimal operational fluid temperature (recall $T_\text {ave}$ = ${40 \pm 0.1}^{\circ }{\textrm{C}}$) across a refined permeability range, while the ground elastic modulus remains constant (*E* = 78MPa). This constant elastic modulus represents a lower bound for partially drained stiff ground, as the elastic modulus significantly influences excess pore water pressures^41^ (Eq. 15). Further details on an element test under varying drainage path conditions are provided in Supplementary Material. Table 4 summarises the resulting thermal loads. For permeabilities below ${9.09}\times {10}^{-15}{\textrm{m}}^{2}$, the thermal load remains constant, exceeding values without hydraulic gradient (Table 3) due to pore fluid composition variations altering the effective thermal conductivity (Eq. 12) and inducing layering effects^42^.

**Table 4:** Thermal loads for seepage analysis with variable intrinsic permeability.

Wall type	*k*, [${\textrm{m}}^{2}$]	Q, [W]
Cantilever	${9.09}\times {10}^{-15}$	1930 (CTemp) 695 (Insu)
	${9.09}\times {10}^{-17}$ ${9.09}\times {10}^{-19}$	1940 700
Milan	${9.09}\times {10}^{-15}$	2650 (CTemp) 606 (Insu)
	${9.09}\times {10}^{-17}$ ${9.09}\times {10}^{-19}$	2660 618
Multi-propped	${9.09}\times {10}^{-15}$	2860 (CTemp) 595 (Insu)
	${9.09}\times {10}^{-17}$ ${9.09}\times {10}^{-19}$	2870 607

**Fig. 7:**
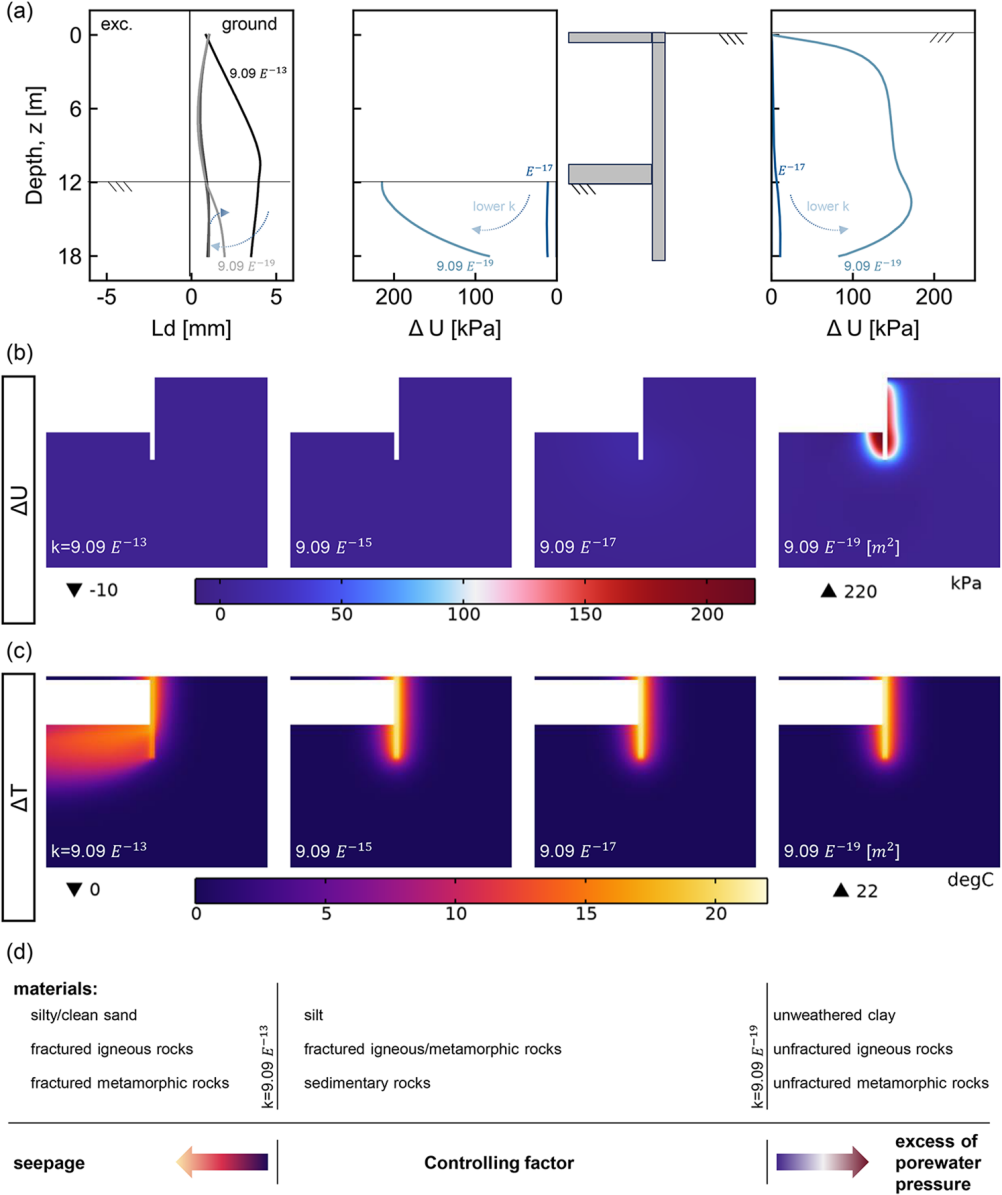
Seepage and thermo-induced pore water pressure effects in Milan walls under perfect insulation. (**a**) Thermo-induced lateral displacement of the pile and distribution of excess pore water pressure ($\Delta U$) in front of and behind the center pile; (**b**) variation of excess pore water pressure with depth; (**c**) temperature increment ($\Delta T$) as a function of intrinsic permeability (*k*); (**d**) materials and intrinsic permeability thresholds governing thermo-hydro-mechanical response. Material classification based on intrinsic permeability ranges proposed by Freeze and Cherry^37^.

Figure 7 examines the development of pore water pressure at the end of heating for different intrinsic permeability values under perfect insulation conditions for the Milan wall. Figure 7 (a) presents the lateral displacements and excess pore water pressure ($\Delta U$) in front of and behind the center pile. Lateral displacements are more pronounced under seepage, but remain relatively constant until permeability threshold values are reached. At this point, excess pore water pressure develops, causing the embedded pile section to pivot about the bottom slab. Excess pore pressures remain below 10KPa at ${9.09}\times {10}^{-17}{\textrm{m}}^{2}$, rising to 220KPa, as permeability decreases, as shown in Fig. 7 (a) and (b), while temperature fields remain consistent (Fig. 7 (c)). Using classifications by Freeze and Cherry^37^, seepage effects develop in silty and clean sands and fractured igneous and metamorphic rocks ($k \ge {9.09}\times {10}^{-13}{\textrm{m}}^{2}$); pore pressure effects develop in unweathered clays and unfractured igneous and metamorphic rocks ($k < {9.09}\times {10}^{-17}{\textrm{m}}^{2}$). These conditions are schematically indicated in Fig. 7 (d).

### Conceptual framework

**Fig. 8:**
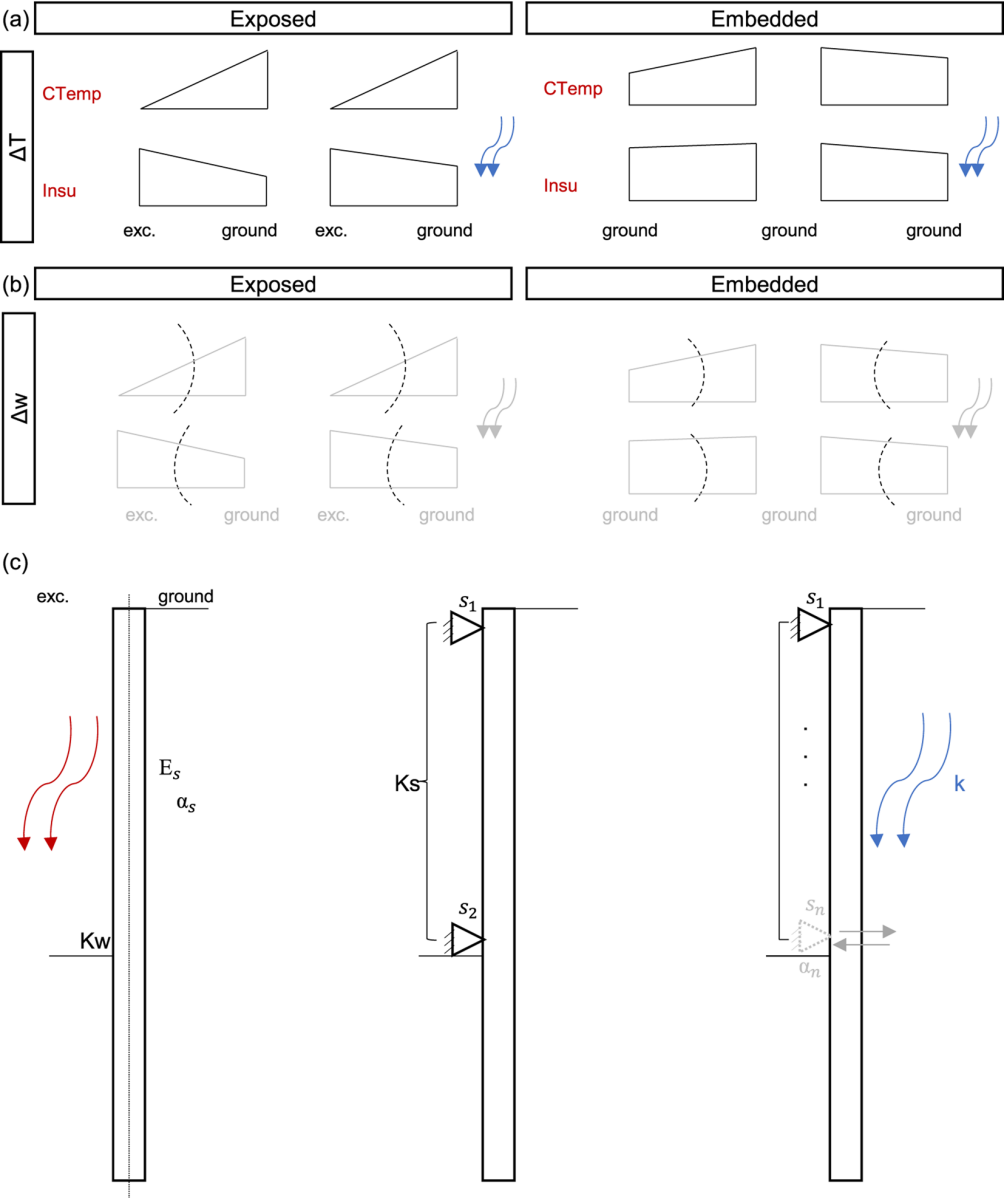
Thermo-hydro-mechanical conceptual framework for energy-piled walls. Schematic representation of: (**a**) temperature increment across the pile cross-section under different thermal boundary conditions and seepage scenarios; (**b**) longitudinal axis curvature at the exposed and embedded pile sections; (**c**) key variables governing the thermo-hydro-mechanical response between ground, energy-piled walls, and lateral supports.

Results are synthesised into a conceptual framework accounting for seepage and pore pressure effects across wall types and ground conditions, as illustrated in Fig. 8. This framework builds upon the thermo-mechanical response of energy-walls descriptions provided by Sailer et al.^22,26^, and thermal gradient flexural effects by Zannin et al.^36^; and extends them to energy piled walls by incorporating seepage, pore water pressure, and lateral supports (i.e., slabs) interaction.

Figure 8(a) represents temperature variation along pile sections under different thermal boundaries and seepage conditions for the exposed and embedded portions of the wall. By the end of heating, the temperature variation is more pronounced under constant temperature than insulation for the exposed sections, diminishing as walls transition into embedded zones, due to the surrounding ground, until becoming minimal. Under seepage, the temperature distribution reverses as water flows and exchanges heat with and around the piles. These temperature distributions along the pile length create vertical displacement ($\Delta w$) gradients across pile cross-sections, producing curvatures along the longitudinal axis, as illustrated in Fig. 8 (b) that explain lateral displacements (recall figures 2, 6), although they are not enough to fully describe the thermo-mechanical response of the piled walls.

A comprehensive thermo-mechanical description requires accounting for ground-wall stiffness variations, seepage influence, and pore pressure presence, as outlined in Fig. 8 (c). Stiffer ground and walls ($K_w$, including support stiffness $K_s$), reduce deformation susceptibility towards the ground under identical temperature distributions. Under seepage ($k \ge {9.09}\times {10}^{-13}{\textrm{m}}^{2}$), thermo-volumetric ground interactions (described by $\alpha _s$) in front of embedded pile sections, along with bottom slab volumetric deformations (in contact with ground, $\alpha _n$), influence wall deformations and increase structural demands. Under low permeability ($k < {9.09}\times {10}^{-17}{\textrm{m}}^{2}$), excess pore pressures (influenced by the solids thermal expansion coefficient per Eq. 15) affect induced displacements in the embedded part. If constrained by a bottom slab, embedded sections may pivot due to unbalanced pore pressures, further increasing thermo-induced structural demands. Together, these variables and the identified permeability thresholds establish a quantitative conceptual framework for energy-piled walls that captures wall-slab-seepage interactions and pore pressure effects, enabling prediction of dominant mechanisms from site ground conditions and wall type.

## Conclusions

This study investigates the thermo-mechanical response of energy-piled walls under varying wall types, pipe layouts, seepage, and thermo-induced pore water pressures. Geothermal, geotechnical, and structural performance are assessed using 3D, time-dependent, coupled finite-element models with variable thermal boundaries and a continuous six-month heating period (cooling of the superstructure). Under the studied conditions, the key findings are:

- The interaction between wall type, thermal boundaries, lateral supports, and wall-ground stiffness influences the thermo-mechanical response of energy-piled walls. Under perfect insulation, lateral displacements are more uniform and larger as the number of lateral supports increases. Under constant temperature, energy-piled walls with more lateral supports experience reduced displacements, while those without supports exhibit larger ones. These variations affect structural demands, increasing localised shear forces and bending moments at lateral support locations, particularly near the pile head and the bottom of the excavation. Generally, stiffer ground and constant temperature conditions result in higher magnitudes.
- A 4U-shaped pipe configuration connected in series is the most favourable arrangement. While 4U-shaped and spiral layouts exhibit similar geothermal and geotechnical performance, the 4U layout results in lower thermo-induced stresses.
- Seepage $(k \ge {9.09}\times {10}^{-13}{\textrm{m}}^{2})$ enhances heat exchange capacity, improving geothermal performance. However, it also increases lateral displacements through wall-bottom slab interaction. Under perfect insulation, this interaction results in higher structural demands, with higher bending moments and shear forces than in non-seepage cases, and inverts the sign of the maximum bending demands direction at the bottom of the excavation.
- Thermo-induced excess pore water pressure $(k < {9.09}\times {10}^{-17}{\textrm{m}}^{2})$ has little effect on lateral displacements but can significantly increase structural demands. In multi-propped walls under constant temperature, bending moments and shear forces may double compared to cases without excess pore pressure.

These findings establish quantitative design guidance for energy-piled walls across diverse ground conditions and construction configurations. The identified permeability thresholds ($k \ge {9.09}\times {10}^{-13}{\textrm{m}}^{2}$ for seepage-dominated; $k < {9.09}\times {10}^{-17}{\textrm{m}}^{2}$ for pore-pressure-dominated behaviour) enable practitioners to anticipate dominant mechanisms during early design stages. The thermo-mechanical response of energy-piled walls is driven not only by thermal gradients across the pile cross-section but also by interactions with lateral supports, thermal boundary conditions, groundwater flow, and excess pore water pressure development. These factors must be carefully considered when evaluating the thermal, geotechnical, and structural performance of energy-piled walls to ensure reliability.

## Supplementary Information

Supplementary Information.

## Data Availability

No raw data has been generated for this study. Numerical simulations were carried out using COMSOL Multiphysics v6.2 (https://www.comsol.com). Data analysis and figure generation were carried out using Jupyter Notebook v6.4.8 (https://www.jupyter.org/). Lateral displacements, axial strains, and shear forces obtained from the models have been made available at Figshare (https://figshare.com/s/a2f6c2254160e92ba72d). Further simulation output data will be made available from the corresponding author on reasonable request.
