# Supplementary material for: Thermo-hydro-mechanical response of energy-piled walls under varying wall configurations, pipe layouts, and seepage conditions
Source: Sci Rep. 2026 Mar 16;16:9198. doi: 10.1038/s41598-026-42923-z (PMC12996308; doi:10.1038/s41598-026-42923-z)
Supplement: Supplementary file 1 — Supplementary Information. [file 41598_2026_42923_MOESM1_ESM.pdf]

Thermo-hydro-mechanical response of energy-piled walls under varying wall configurations, pipe layouts, and seepage conditions

Luis Villegas *[1,2], Guillermo Narsilio[1], and Raul Fuentes[2]

[1]Department of Infrastructure Engineering, The University of Melbourne, Parkville, Australia
[2]Institute of Geomechanics and Underground Technology, RWTH Aachen University, Aachen, Germany

*villegas-negrette@gut.rwth-aachen.de,luis.villegas@unimelb.edu.au

A Supplementary Material

This supplementary material provides: (i) validation of the thermo-hydro-mechanical coupling (Figure A.1); (ii) thermal load determination methodology (Figure A.2); (iii) mesh sensitivity analysis (Figure A.3); (iv) extended pipe layout effects (Figure A.4); and (v) thermal expansion coefficient influence on stress evolution (Figure A.5).

Analytical

$$\Delta U_j = \frac{3n_{j-1}(\propto_{m,f} - \propto_{m,s})\Delta T}{\frac{n_{j-1}}{\mathrm{k}_{m,f}} + \frac{3(1-2v)}{E_s}}$$

$$U_j = \Delta U_j + U_{j-1}$$

$$\Delta e_j = \frac{3(1-2v)(1+e_{j-1})\Delta U_{j-1}}{E_s}$$

$$e_j = \Delta e_j + e_{j-1}$$

$$n_j = \frac{e_j}{1+e_j}$$

Parameters

Property	Value
n, []	0.44
E_s, [MPa]	200
v, []	0.3
$\propto_{m,s}$, [με/K]	20
$\propto_{m,f}$ [με/K]	0.1
$\mathrm{k}_{m,f}$ [MPa]	2200

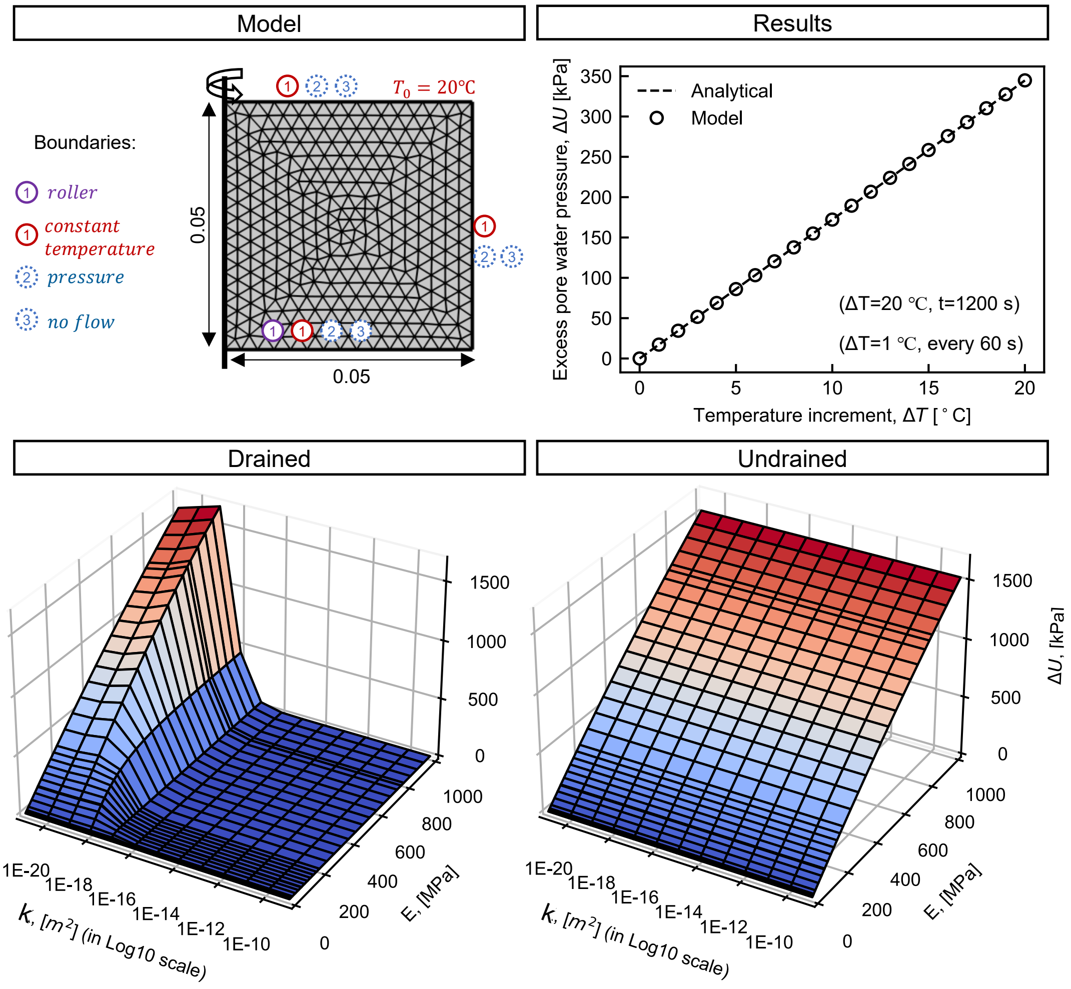

Fig. A.1. Validation of thermo-hydro-mechanical coupling for thermo-induced pore water pressure prediction. Comparison between numerical results (COMSOL Multiphysics) and analytical solution proposed by Cui 2015 for a thermally loaded saturated soil element under undrained conditions. The element is subjected to a temperature increment of $\Delta T = 20°\mathrm{C}$ applied over 20 minutes. Results are shown for different combinations of ground permeability ($k = 10^{-10}$ to 10^{-20} m^2) and stiffness ($E = 0$ to 1000 MPa), demonstrating that excess pore water pressure development is primarily controlled by drainage conditions and elastic modulus. Close agreement between numerical and analytical solutions validates the implementation of governing equations for coupled thermo-hydro-mechanical analysis.

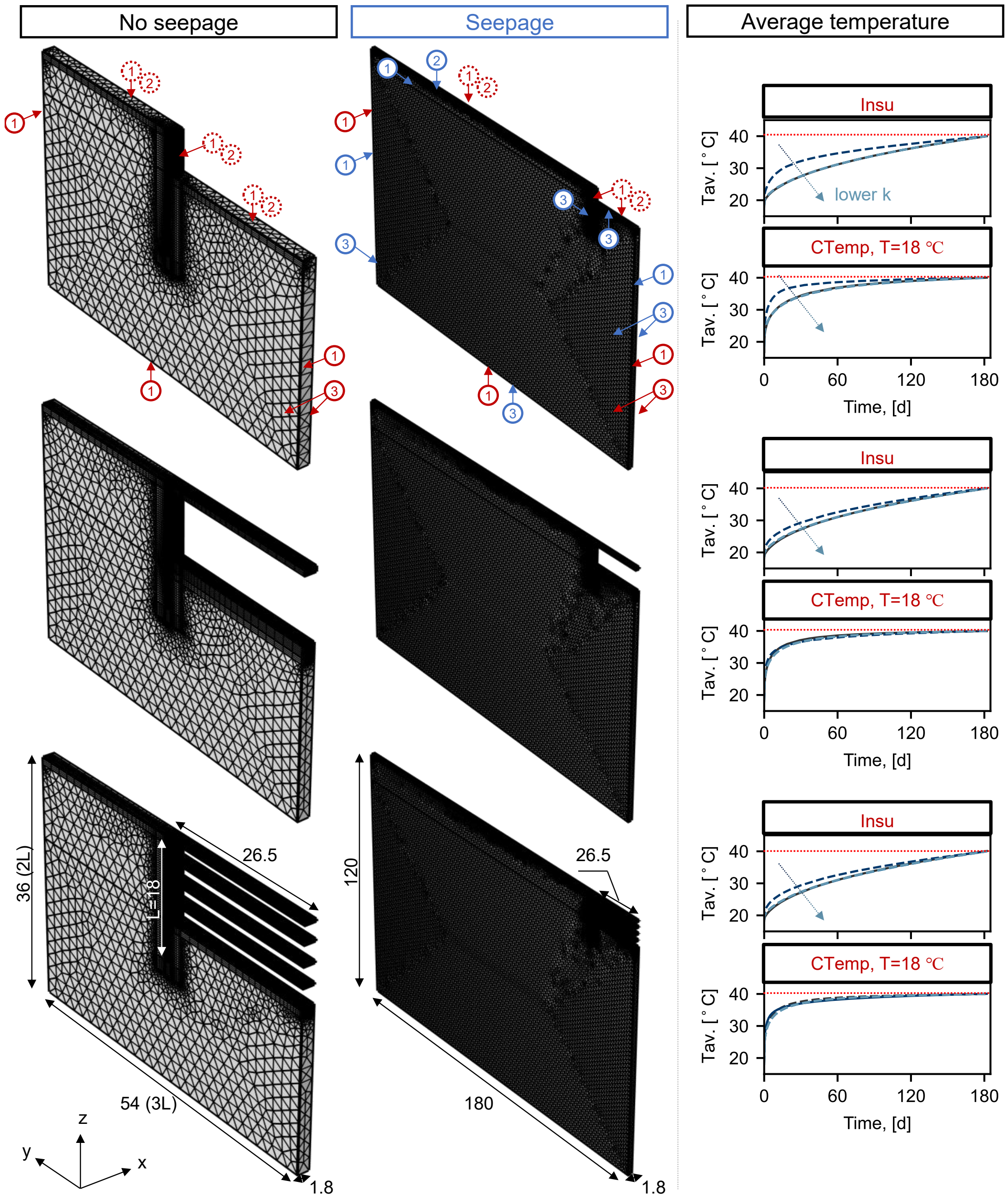

Fig. A.2. Thermal load determination methodology for energy-piled walls. Representative wall sections used for thermal load analysis under no seepage (left) and seepage (center) conditions, showing geometric configuration, pile-to-pile spacing, and boundary conditions. Evolution of carrier fluid average temperature over the six-month heating period for different wall configurations under constant temperature and perfect insulation boundary conditions (right). For each case, the thermal load per pile is chosen to reach $T_{ave} = 40 \pm 0.1$°C throughout the analysis period, representing optimal operational conditions. The resulting thermal loads are summarised in Tables 3 and 4.

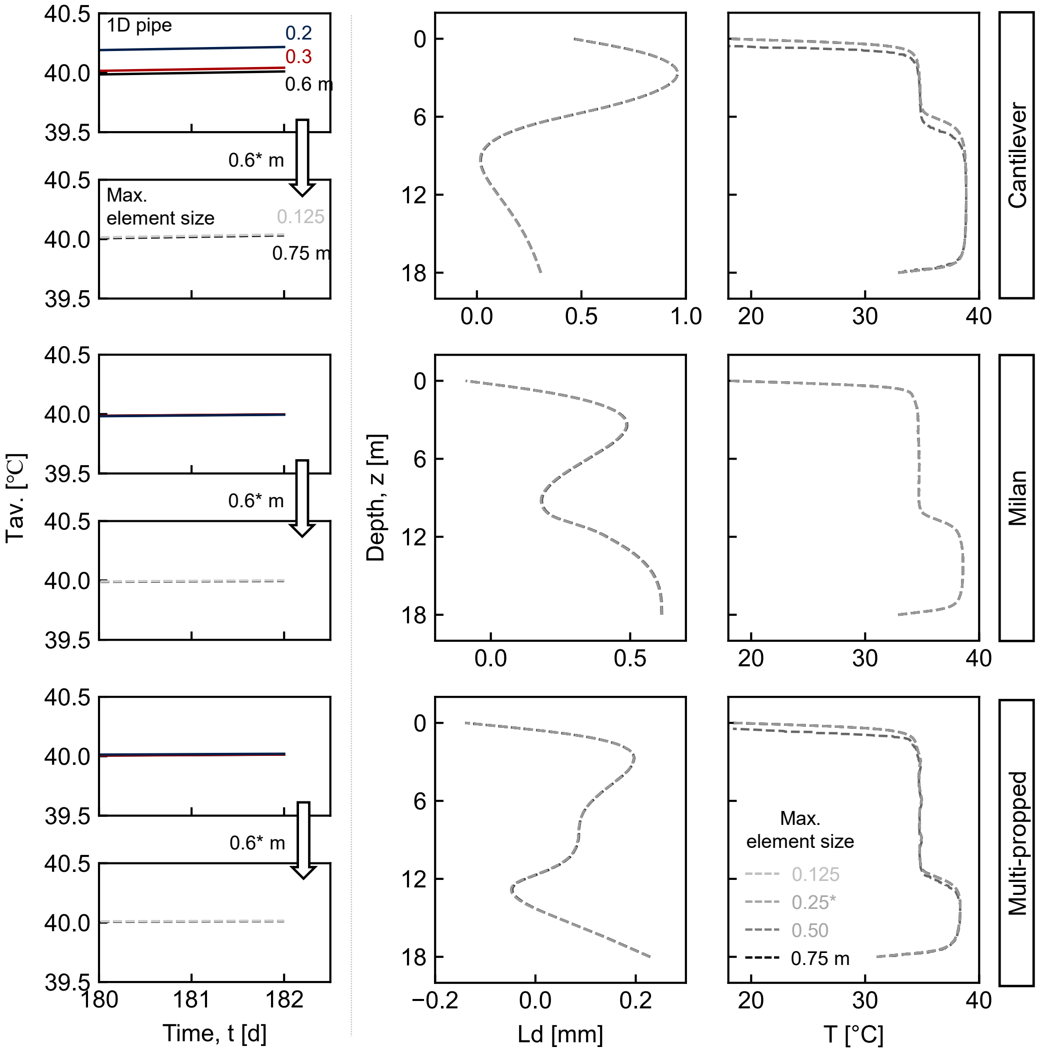

Fig. A.3. Mesh sensitivity analysis for different wall configurations under constant temperature boundary conditions. Convergence assessment for: average temperature in 1D pipe elements (left), thermo-induced pile lateral displacement (centre), and pile temperature along its axis (right). Progressively refined meshes were analysed with maximum element sizes ranging from 0.75 to 0.125 m for both the wall/near-field domains and 1D pipe elements. Results demonstrate negligible differences between the finest mesh and the adopted mesh (*): 0.6 m for 1D pipe elements, 0.25 m for wall and refined near-field domains.

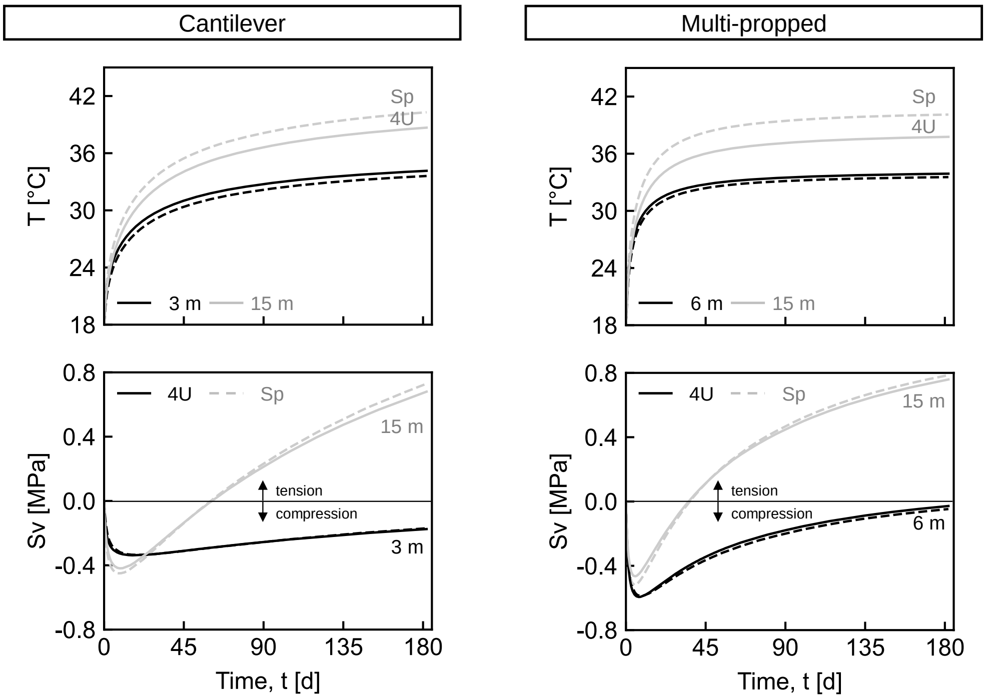

Fig. A.4. Pipe layout influence on temperature and stress evolution in the stiffest ground (E = 780 MPa) under constant temperature boundary conditions. Cantilever wall (left); multi-propped wall (right). Time-dependent volume-averaged temperature (top row) and vertical thermo-induced stress (bottom row) within 2 m segments centred at representative depths: 3 m (cantilever only), 6 m (multi-propped only), and 15 m (both walls). Results compare 4U-shaped loops (4U) and spiral (Sp) configurations. These findings complement Figure 5, demonstrating consistent pipe layout effects across all wall types.

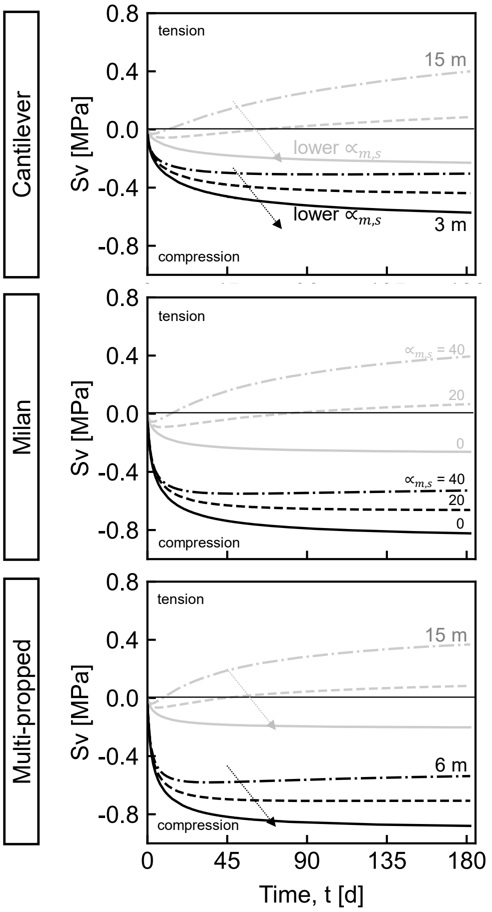

Fig. A.5. Coefficient of thermal expansion (α_s) effect on thermo-induced vertical stress evolution in different wall configurations, under constant temperature boundary conditions. Time-dependent volume-averaged vertical stress within 2 m segments centered at representative depths: 3 m, 6 m (exposed section), and 15 m (embedded section). Ground elasticity modulus E = 78 MPa; thermal expansion coefficients: α_s = 0, 20, 40 $\mu\epsilon$/K. These findings complement Figure 5 by showing the coefficient of thermal expansion effect across all wall types at medium ground stiffness.
